# Safety and Tolerability of Serdexmethylphenidate/Dexmethylphenidate Capsules in Children with Attention-Deficit/Hyperactivity Disorder: A 12-Month, Open-Label Safety Study

**DOI:** 10.1089/cap.2022.0076

**Published:** 2023-03-20

**Authors:** Ann C. Childress, Andrea Marraffino, Andrew J. Cutler, Charles Oh, Matthew N. Brams

**Affiliations:** ^1^ Center for Psychiatry and Behavioral Medicine, Las Vegas, Nevada, USA.; ^2^ Accel Research Sites Network, Maitland, Florida, USA.; ^3^ SUNY Upstate Medical University, Syracuse, New York, USA.; ^4^ Neuroscience Education Institute, Lakewood Ranch, Florida, USA.; ^5^ Corium, LLC, Boston, Massachusetts, USA.; ^6^ Bayou City Research, Houston, Texas, USA.

**Keywords:** ADHD-RS-5, CGI-S, dexmethylphenidate, safety, serdexmethylphenidate, SDX/d-MPH

## Abstract

**Objective:**

Serdexmethylphenidate/dexmethylphenidate (SDX/d-MPH) is approved for the treatment of patients aged ≥6 years with attention-deficit/hyperactivity disorder (ADHD). A pivotal double-blind (DB) study of children aged 6–12 years with ADHD demonstrated efficacy for ADHD with good tolerability. In this study, we assessed the safety and tolerability of daily oral SDX/d-MPH for up to 1 year in children with ADHD.

**Methods:**

This was a dose-optimized, open-label safety study with SDX/d-MPH in children aged 6–12 years with ADHD that included subjects who successfully completed the DB study (rollover) and new subjects. The study consisted of a 30-day screening phase, a dose optimization phase for new subjects only, a 360-day treatment phase, and follow-up. Adverse events (AEs) were assessed from the first day of SDX/d-MPH administration to the end of the study. During the treatment phase, ADHD Rating Scale-5 (ADHD-RS-5) and Clinical Global Impressions–Severity (CGI-S) scale assessments were used to evaluate ADHD severity.

**Results:**

Of the 282 subjects enrolled (70 rollover; 212 new), 28 discontinued treatment in the dose optimization phase and 254 entered the treatment phase. By study completion, 127 had discontinued and 155 had completed the study. The treatment-phase safety population included all enrolled subjects who received ≥1 dose of study drug and had ≥1 postdose safety assessment. Of 238 subjects assessed in the treatment-phase safety population, 143 (60.1%) had ≥1 treatment-emergent adverse events (TEAEs), and 36 (15.1%), 95 (39.9%), and 12 (5.0%) had mild, moderate, or severe TEAEs, respectively. The most common TEAEs were decreased appetite (18.5%), upper respiratory tract infection (9.7%), nasopharyngitis (8.0%), decreased weight (7.6%), and irritability (6.7%). There were no clinically meaningful trends in electrocardiograms, cardiac events, or blood pressure events, and none led to discontinuation. Two subjects had eight serious AEs that were unrelated to treatment. There were overall reductions in ADHD symptoms and severity as assessed by ADHD-RS-5 and CGI-S during the treatment phase.

**Conclusions:**

In this 1-year study, SDX/d-MPH was found to be safe and well tolerated and comparable with other methylphenidate products, with no unexpected safety findings. SDX/d-MPH also showed sustained efficacy during the 1-year treatment period. ClinicalTrials.gov identifier: NCT03460652.

## Introduction

Methylphenidate (MPH) has been in use for the treatment of attention-deficit/hyperactivity disorder (ADHD) for more than a half century. It is the most commonly prescribed medication for the treatment of patients with ADHD because of its effects on mitigating the core symptoms of ADHD in children and its overall favorable safety record (Storebø et al, 2018).

Serdexmethylphenidate/dexmethylphenidate (SDX/d-MPH; Azstarys^®^) is approved for the treatment of patients aged ≥6 years with ADHD. SDX/d-MPH contains a fixed molar ratio of 70% SDX, a novel prodrug of d-MPH, and 30% d-MPH. After oral intake of an SDX/d-MPH capsule, early exposure to MPH is governed primarily by d-MPH in the formulation, and mid- to late-day exposure is governed by the gradual conversion of SDX to d-MPH (Kollins et al, 2021). SDX is designed to be pharmacologically inactive until it is gradually converted to active d-MPH in the lower intestinal tract. The recommended daily starting dose of SDX/d-MPH is 39.2/7.8 mg (30 mg molar equivalent of total d-MPH HCl). After 1 week, daily dose can be adjusted based on efficacy and tolerability by increasing to 52.3/10.4 mg or decreasing to 26.1/5.2 mg daily (40 and 20 mg molar equivalents, respectively, of total d-MPH HCl).

Results of a pivotal, 1-month, randomized, placebo-controlled, double-blind (DB), dose-optimized, laboratory classroom study of children aged 6–12 years with ADHD demonstrated that SDX/d-MPH was well tolerated, with adverse events (AEs) that were comparable with those of other stimulant treatments (Kollins et al, 2021). In addition, significant improvements in ADHD symptoms were seen versus placebo in children aged 6–12 years.

The safety analysis of the pivotal study showed that 67% of subjects experienced at least one treatment-emergent adverse event (TEAE) during the dose optimization (DO) phase, and 22.7% of subjects experienced at least one TEAE during the DB phase. TEAEs were mostly mild (56.8%) to moderate (29.7%); 3.2% had severe TEAEs. There were no serious adverse events (SAEs) in either phase. The most common AEs occurring in ≥2% of subjects during the DO phase were decreased appetite (24.5%), insomnia (15.5%), affect lability (11.6%), upper abdominal pain (9.7%), headache (7.7%), and irritability (7.7%).

During the treatment phase, the most common TEAEs were headache (5.4%), upper abdominal pain (4.1%), and insomnia and pharyngitis (both 2.7%) (Kollins et al, 2021). Overall, the findings from the 1-month pivotal study showed that SDX/d-MPH has a favorable AE profile that is comparable with that of other MPH-based treatments. The rationale for the current study was to investigate the safety and tolerability of daily SDX/d-MPH use for a 1-year duration and to determine if effectiveness of SDX/d-MPH was sustained in treating children with ADHD.

## Methods

This was a DO, open-label safety study with SDX/d-MPH administered orally in children aged 6–12 years with ADHD (NCT03460652). The study was conducted at 18 sites in the United States. The study protocol and amendments were approved by an institutional review board before each center's study initiation. Written informed consent was obtained from all subjects before enrollment in the study. The first subject was screened on February 21, 2018, and the last follow-up visit was on June 27, 2019. This study included new subjects and those who completed the pivotal DB, placebo-controlled laboratory classroom study and were rolled over into the current study (rollover subjects).

### Subjects

Subjects who had not participated in the previous laboratory classroom study were required to be at least 6 years and <13 years of age at the start of the DO phase in the present study. Subjects rolling over from the laboratory classroom study were included if they had reached 13 years of age by the time they entered into the present study. All subjects had to be in overall good health without any clinically relevant abnormalities determined by physical and neurological examinations, vital signs, electrocardiograms (ECGs), medical history, and clinical laboratory values. New subjects must have had a body weight of ≥21 kg at screening. The rollover subjects had the same body weight requirements when enrolling in the earlier pivotal study. At least one parent or legal guardian of the subject had to give voluntary written permission to participate in the study, and the subject had to give written or oral approval before participating in the study.

New subjects had to meet *Diagnostic and Statistical Manual of Mental Disorders, Fifth Edition* (American Psychiatric Association, 2013), criteria for a primary diagnosis of ADHD (combined, inattentive, or hyperactive/impulsive presentation) per clinical evaluation and confirmed by the Mini International Neuropsychiatric Interview for Children and Adolescents. New subjects also had to have a score of at least 3 (mildly ill) on the clinician-administered Clinical Global Impressions–Severity (CGI-S) scale at the start of the DO phase or after medication washout, if applicable.

Subjects were excluded if they had any diagnosis of bipolar I or II disorder, major depressive disorder, conduct disorder, or obsessive-compulsive disorder or any history of psychosis, autism spectrum disorder, disruptive mood dysregulation disorder, intellectual disability, Tourette syndrome, or confirmed genetic disorder with cognitive and/or behavioral disturbances. Subjects with oppositional defiant disorder were permitted to enroll in the study provided that it was not the primary focus of treatment and, in the opinion of the investigator, was mild to moderate and as long as eligible subjects with oppositional defiant disorder were appropriate and cooperative during screening.

### Study design

The study design consisted of a 30-day screening phase, a DO phase (for new subjects), a 360-day treatment phase, and a follow-up visit. Subjects who successfully completed the laboratory classroom study were rolled over into the current trial within 45 days of their last dose of SDX/d-MPH in the previous study. The rollover subjects bypassed the screening and DO phases and continued into the 360-day treatment phase. These rollover subjects were entered into this trial on the same day as their follow-up visit in the previous study or up to 45 days later. The starting dose for these subjects in the treatment phase was the same as their optimized dose from the previous study.

New subjects were defined as those who did not participate in the previous study or entered the trial >45 days after their last dose in the previous study. New subjects underwent screening. For both rollover and new subjects, the dose of SDX/d-MPH could be changed based on their individual response and tolerability to the treatment.

During the DO phase (for new subjects only), subjects titrated to their optimized dose based on their individual tolerability and best response to the treatment in the opinion of the investigator. New subjects started treatment with 39.2/7.8 mg SDX/d-MPH daily for 7 days. Dose adjustments, if needed, were performed at approximately weekly intervals. The investigator evaluated the subject's therapeutic response and tolerability to treatment and decided whether the current SDX/d-MPH dose should be increased, decreased, or remain the same for the next week of dosing. The dose at the end of the third week was assigned as the optimized dose consisting of 26.1/5.2, 39.2/7.8, or 52.3/10.4 mg SDX/d-MPH daily. The starting dose during the treatment phase was the optimized SDX/d-MPH dose at the end of the DO phase. During the treatment phase, all subjects continued their optimized dose of SDX/d-MPH daily.

### Safety assessments

All baseline and safety data were analyzed using the safety population. The safety population included all enrolled subjects who received at least one dose of study medication and had at least one postdose safety assessment.

AEs were assessed and recorded from the first day of SDX/d-MPH administration through the end of the study through either follow-up or early termination. Both rollover and new subjects completed a follow-up visit ∼3 days after the last dose of SDX/d-MPH. A complete medical history was obtained at the screening visit. Physical examinations, clinical laboratory assessments, and ECGs were performed at screening, after ∼6 months of treatment, and at the end of the treatment phase. Vital signs (sitting blood pressure, pulse rate, respiratory rate, and oral temperature) were assessed at each study visit.

For safety assessments, descriptive statistics were reported for continuous and categorical data. *Z*-score was used to analyze a subject's weight and height against a reference population using the United States 2000 Centers for Disease Control Growth Charts (ages 2 to <20 years) as the reference (Kuczmarski et al, 2002). The modified abbreviated Children's Sleep Habits Questionnaire (CSHQ) was used to assess sleep behavior during the treatment phase. Of a total score of 99, the clinical cutoff indicating the presence of sleep disorders is a CSHQ score of ≥41. A reduction in CSHQ scores indicates improvement in sleep (Owens et al, 2000).

### Efficacy assessments

All efficacy analyses and CSHQ assessments were analyzed using the efficacy population. This population included all enrolled subjects who received at least 30 days of study medication in the treatment phase, who had adequate data to assess the change from baseline of the efficacy parameters, and who had no protocol deviations that could affect the efficacy parameters.

For new subjects, during the DO phase, the ADHD Rating Scale-5 (ADHD-RS-5), CGI-S scale, and Clinical Global Impressions–Improvement scale were used to guide DO in conjunction with tolerability and safety (Busner and Targum, 2007; DuPaul et al, 2016). ADHD-RS-5 is an 18-item scale based on *Diagnostic and Statistical Manual of Mental Disorders, Fifth Edition* (American Psychiatric Association, 2013) criteria of ADHD that rates symptoms on a 4-point scale, ranging from 0 (no symptoms) to 3 (severe or frequent symptoms) (DuPaul et al, 2016).

The CGI-S is a clinician-rated scale that measures the severity of psychopathology (ADHD symptoms in the study) on a scale from 1 (not at all ill) to 7 (among the most extremely ill) (Busner and Targum, 2007). During the treatment phase, the ADHD-RS-5 and CGI-S scale assessments were used to evaluate the changes in ADHD symptoms and severity over time and could be used in conjunction with tolerability and safety to adjust the dose of SDX/d-MPH. Wilcoxon signed-rank test was used to assess the significance of changes from baseline through follow-up visits (significance level of 0.05). A last-observation-carried-forward approach was used for missing data.

## Results

### Subject disposition and dosing

**FIG. 1.**
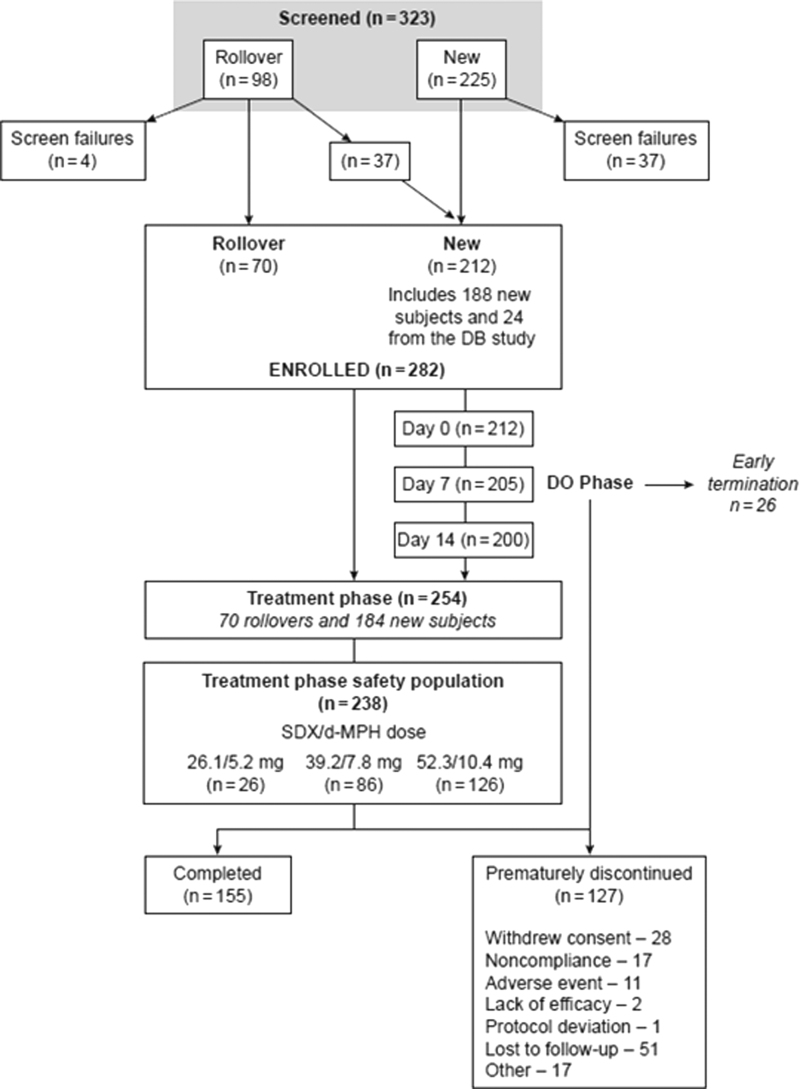
Subject disposition. DB, double-blind; DO, dose optimization; SDX-d-MPH, serdexmethylphenidate/dexmethylphenidate.

Of the 323 subjects screened, 282 were enrolled in the study, of whom 70 were rollover subjects and 212 were new subjects (including 24 enrolled as new subjects from the previous study because their last completed dose in the previous study was >45 days before the first dose in the current study; Fig. 1). Of the 282 enrolled subjects, 254 entered the treatment phase, which included 70 rollover subjects and 184 new subjects. Of the 282, 155 subjects completed treatment and 127 discontinued early. Reasons for discontinuation were lost to follow-up (*n* = 51, 18.1%), withdrew consent (*n* = 28, 9.9%), noncompliance (*n* = 17, 6.0%), AEs (*n* = 11, 3.9%), lack of efficacy (*n* = 2, 0.7%), protocol deviation (*n* = 1, 0.4%), and other (*n* = 17, 6.0%). Two hundred thirty-eight subjects were in the treatment-phase safety population.

On day 0, the mean (standard deviation [SD]) daily dose of d-MPH HCl equivalent was 32.54 mg (7.28 mg), or 0.94 mg/kg (0.35 mg/kg) of body weight. At 6 months of treatment, the mean daily dose of d-MPH HCl equivalent was 33.56 mg (7.11 mg), or 0.94 mg/kg (0.36 mg/kg). At the last dispensing visit, day 330, the mean daily dose of d-MPH HCl equivalent was 35.08 mg (6.38 mg), or 0.95 mg/kg (0.34 mg/kg) of body weight.

### Baseline and demographic characteristics

The mean age of subjects was 9.1 years, and most (61%) were male (Table 1). Nineteen percent were of Hispanic or Latino ethnicity. The racial makeup of the subjects was predominantly White (48%) and Black/African American (47%). At baseline, subjects in the efficacy population had a mean (SD) ADHD-RS-5 score of 41.5 (7.7) and a mean CGI-S score of 4.7 (0.7). The mean (SD) score on the CSHQ at baseline was 53.5 (6.0).

**Table 1.** Subject Demographics and Baseline Characteristics

Parameter	Subjects (*N* = 238)
Age, years	9.1 (1.87)
Sex, *n* (%)	
Male	145 (60.9)
Female	93 (39.1)
Ethnicity, *n* (%)	
Hispanic or Latino	45 (18.9)
Not Hispanic or Latino	193 (81.1)
Race, *n* (%)	
White	113 (47.5)
Black/African American	111 (46.6)
Multiracial	9 (3.8)
Asian	2 (0.8)
Other	2 (0.8)
American Indian/Alaska Native	1 (0.4)
Weight, kg	38.6 (13.9)
Height, cm	139.6 (11.9)
Body mass index, kg/m^2^	19.3 (4.6)
ADHD-RS-5, overall score	41.5 (7.7)
CGI-S	4.7 (0.7)
CSHQ total score	53.5 (6.0)

Values shown are mean (SD), unless otherwise noted.

ADHD-RS-5, Attention-Deficit/Hyperactivity Disorder Rating Scale-5; CGI-S, Clinical Global Impressions–Severity; CSHQ, Children's Sleep Habits Questionnaire; SD, standard deviation.

### Safety results

In the DO phase (safety population, *n* = 208), 113 subjects (54.3%) experienced at least one TEAE (Table 2). The most common TEAEs in the DO phase were decreased appetite (18.8%), followed by insomnia and irritability (both 6.7%), and initial insomnia (5.3%) (Table 2). In the treatment phase (safety population, *n* = 238), 26 subjects were optimized to 26.1/5.2 mg, 86 subjects were optimized to 39.2/7.8 mg, and 126 subjects were optimized to 52.3/10.4 mg SDX/d-MPH daily (Fig. 1). Of the 238 subjects in the treatment phase, 143 (60.1%) experienced at least one TEAE, and 36 (15.1%) experienced mild TEAEs, 95 (39.9%) experienced moderate TEAEs, and 12 (5.0%) experienced severe TEAEs (Table 2). The most common TEAEs in the treatment phase were decreased appetite (18.5%), upper respiratory tract infection (9.7%), nasopharyngitis (8.0%), decreased weight (7.6%), and irritability (6.7%). There were no life-threatening or fatal TEAEs reported.

**Table 2.** Treatment-Emergent Adverse Events During the Dose Optimization and Open-Label Treatment for Up to 12 Months (Safety Population)

	DO phase (*N* = 208)	Treatment phase (*N* = 238)
Subjects with ≥1 TEAE	113 (54.3)	143 (60.1)
Mild	56 (26.9)	36 (15.1)
Moderate	53 (25.5)	95 (39.9)
Severe	4 (1.9)	12 (5.0)
Life-threatening	0	0
Fatal	0	0
TEAEs ≥5% incidences Preferred term		
Decreased appetite	39 (18.8)	44 (18.5)
Upper respiratory tract infection		23 (9.7)
Nasopharyngitis		19 (8.0)
Decreased weight		18 (7.6)
Insomnia	14 (6.7)	12 (5.0)
Irritability	14 (6.7)	16 (6.7)
Initial insomnia	11 (5.3)	
Increased weight		12 (5.0)

Values shown are *n* (%).

DO, dose optimization; TEAE, treatment-emergent adverse event.

All AEs leading to discontinuation of study drug during the DO phase and treatment phase were classified as TEAEs. During the DO phase, four subjects (1.9%) with at least one TEAE discontinued treatment because of an AE. TEAEs leading to treatment discontinuation in the DO phase were aggression, irritability, psychotic disorder, and nausea (each *n* = 1). Irritability, psychotic disorder, and nausea were considered related to the study drug by the investigators. Six subjects (2.5%) discontinued treatment because of eight TEAEs during the treatment phase. Reasons for discontinuation included incidences of initial insomnia (*n* = 2), irritability (*n* = 2), depression (*n* = 1), and suicidal ideation (*n* = 1), followed by incidences of leukopenia and decreased appetite (each *n* = 1). Two of the six subjects discontinued because of two TEAEs, one was discontinued for irritability and decreased appetite, and another was discontinued for depression and suicidal ideation. All treatment-phase discontinuations were assessed as being related to study drug.

Two subjects experienced a total of eight SAEs during the treatment phase. One subject had seven SAEs because of preexisting conditions of asthma and steroid-induced diabetes mellitus, and another subject experienced a single seizure that was unrelated to treatment with SDX/d-MPH.

TEAEs related to vital sign assessments in the DO phase were increased blood pressure (two subjects [1.0%]), increased heart rate (one subject [0.5%]), and tachycardia (four subjects [1.9%]); and in the treatment phase, vital sign-related TEAEs were increased blood pressure (two subjects [0.8%]), increased diastolic blood pressure (one subject [0.4%]), tachycardia (two subjects [0.8%]), and sinus tachycardia (one subject [0.4%]). A total of four subjects experienced seven laboratory-related TEAEs during the study in the treatment phase related to severe hyperglycemia, mild increased alkaline phosphatase, mild increased blood potassium, moderate decreased neutrophil and white blood cell counts, leukopenia, and high glucose level.

Overall, there were no clinically meaningful trends in ECG parameters over time, and no clinically significant ECG abnormalities were reported. One subject had a dose reduction implemented because of palpitations.

The mean body weight (SD) at baseline was 38.6 kg (13.9 kg), and after ∼12 months of treatment, the mean body weight was 41.1 kg (14.6 kg); a mean change from baseline weight of +3.4 kg (4.3 kg). Body weight decreased in 18 subjects (7.6%) and increased in 12 subjects (5.0%) during the treatment phase. The mean weight *Z*-score (SD) decreased from a baseline score of 0.74 (1.13) to 0.51 (1.08) 12 months after treatment; a mean decrease from baseline score of −0.20 (0.50). The mean height (SD) at baseline was 139.6 cm (11.9 cm). After ∼12 months of treatment, the mean height was 143.4 cm (11.8 cm); a mean change from baseline height of +4.9 cm (2.5 cm). The mean height *Z*-score decreased from a baseline score of 0.54 (0.98) to 0.24 (0.96) 12 months after treatment; a mean decrease from baseline score of −0.21 (0.39). With regard to sleep assessment, the mean CSHQ score improved while on treatment, with a mean score (SD) of 53.5 (6.0) at baseline and decreasing significantly to 49.1 (4.7; *p* < 0.001) after 12 months of treatment.

### Efficacy results

**FIG. 2.**
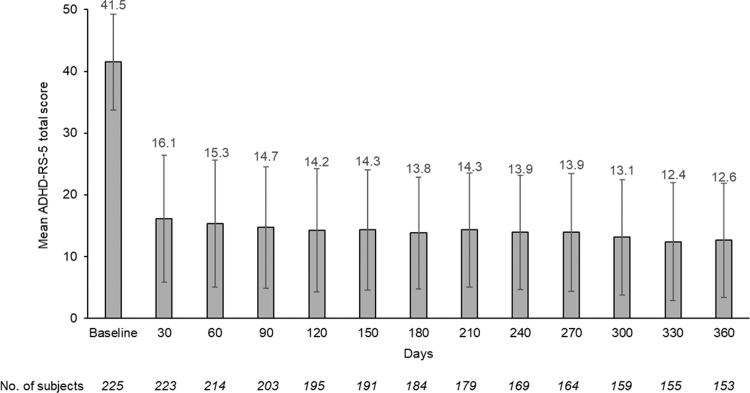
Mean ADHD-RS-5 scores by visit (treatment phase, efficacy population). Bars are standard deviations. ADHD-RS-5, Attention-Deficit Hyperactivity Disorder Rating Scale-5.

**FIG. 3.**
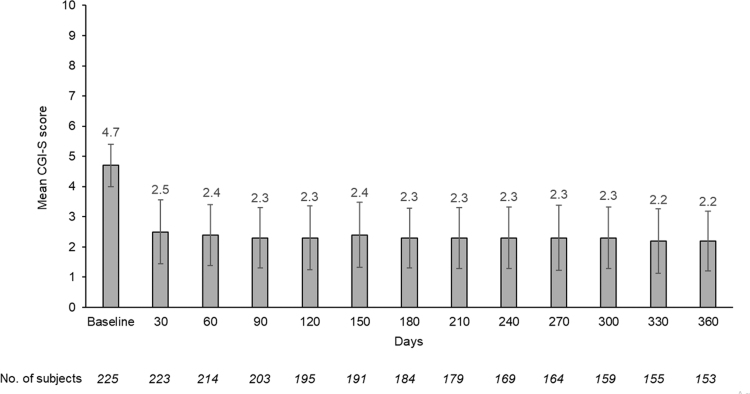
Mean CGI-S scores by visit (treatment phase, efficacy population). Bars are standard deviations. CGI-S, Clinical Global Impressions–Severity.

ADHD symptoms, as measured by ADHD-RS-5 scores, decreased (improved) markedly after 1 month of treatment from a mean (SD) score of 41.5 (7.7) at baseline to 16.1 (10.3; Fig. 2), a change from baseline score of −25.3 (12.1; *p* < 0.001), and the mean score stabilized in the 12–15 range thereafter for the remainder of the study, with an ∼70% reduction at 12 months. There was a statistically significant improvement in the clinical severity of ADHD as assessed by the CGI-S rating scale. After 1 month of treatment, the CGI-S score decreased from a mean (SD) score of 4.7 (0.7) at baseline to 2.5 (1.1), a change from baseline score of −2.2 (1.1; *p* < 0.0001), and the mean score remained in the 2.2–2.4 range for the remainder of the study (Fig. 3).

## Discussion

This 1-year DO, open-label safety study to evaluate the long-term safety and tolerability of SDX/d-MPH is the second phase 3 clinical trial with SDX/d-MPH to be completed in children aged 6–12 years with ADHD, after the pivotal, short-term DB, laboratory classroom study (Kollins et al, 2021). Rollover subjects continued from that pivotal study at their optimized dose, and new subjects were also enrolled after DO. Boys predominated in this study, and the racial makeup of the children was mostly White or Black/African American, which is consistent with prevalence estimates of the demographic makeup of children diagnosed with ADHD (Danielson et al, 2018). Additionally, 19% of children were of Hispanic or Latino ethnicity, which is likely reflective of the location of the study centers in states such as Colorado, Nevada, Texas, and Florida with large Hispanic or Latino populations.

This 1-year study showed that SDX/d-MPH was safe and well tolerated. The most common TEAEs were decreased appetite, irritability, decreased weight, insomnia, upper respiratory tract infection, and nasopharyngitis. There were no new or unexpected TEAEs compared with those of the pivotal study that could potentially be attributed to SDX/d-MPH. Because MPH products are associated with increased blood pressure and heart rate, which are risk factors for cardiovascular morbidity (Hennissen et al, 2017), both were monitored continuously during this study. The incidence of TEAEs associated with vital signs, including elevated blood pressure and increased heart rate, was low (≤1% of subjects), and there were minimal changes in vital sign measurements overall.

ECGs did not show prolongation of QT interval. No subjects discontinued treatment because of increased blood pressure or heart rate. Overall effects of SDX/d-MPH on reductions in body weight and height were consistent with those observed with other MPH products (Faraone and Giefer, 2007; Spencer et al, 2006). Assessment of sleep using the CSHQ showed improvement in sleep over the course of the study, and more importantly, there was no worsening in sleep problems with SDX/d-MPH treatment. This is important because many patients with ADHD have sleep disturbances at baseline before starting ADHD treatment.

In the treatment phase, 60% of subjects reported ≥1 AE, and 2.5% of subjects discontinued treatment. Placed into context with other MPH products for ADHD, the percentages of AEs with SDX-d-MPH were equivalent or fewer than those reported with other treatments. In general, there were fewer study discontinuations due to AEs with SDX/d-MPH than with those reported in other MPH studies.

In a comprehensive 2018 systematic review of AEs associated with MPH, much higher rates of AEs were found compared with those in the present study. In 177 noncomparative studies in children and adolescents (aged 3–20 years) involving 2,207,751 subjects with ADHD, the incidence of non-serious AEs was 51.2% (range, 41.2%–61.1%), with SAEs ranging from 0.7% to 2.0% (Storebø et al, 2018). Withdrawal of MPH because of non-serious AEs ranged from 4.8% to 7.9%, and withdrawal because of SAEs ranged from 0.6% to 2.3% (Storebø et al, 2018).

In a 1-year study of the osmotic-release oral system (OROS) formulation of MPH in children aged 6–13 years with ADHD, 344 of 407 subjects (84.5%) reported ≥1 AE, and 6.9% of subjects discontinued the medication because of an AE (Wilens et al, 2003). Headache (25%) was the most commonly reported AE. With SDX/d-MPH, the percentage of subjects reporting headache was 4.6%. Other AEs reported with OROS-MPH in ≥5% of subjects included insomnia (14.7%), appetite suppression (13.5%), and abdominal pain and twitching (each 7.6%).

In a 1-year study of an MPH multilayer release (MPH-MLR) extended-release formulation in children aged 4–6 years with ADHD, 65 of 89 subjects (73%) experienced TEAEs, and 11.2% discontinued treatment because of an AE (Childress et al, 2022). The most commonly reported AE was decreased weight in 18% of subjects. With SDX/d-MPH, 7.6% of subjects had decreased weight. Other AEs reported with MPH-MLR, including decreased appetite (18.0%), upper respiratory tract infection (9.0%), nasopharyngitis (11.2%), insomnia (9.0%), and irritability (7.9%), were comparable with those for SDX/d-MPH.

In this study, efficacy was assessed by ADHD-RS-5 and CGI-S scores. SDX/d-MPH treatment produced a noticeable improvement in ADHD-RS-5 and CGI-S scores within 1 month of starting treatment, and continued effectiveness was seen during the 1-year treatment period, as shown by sustained low ADHD-RS-5 and CGI-S scores from baseline.

Limitations of this study include the open-label nature of the study design and the lack of placebo or a comparator product. There may also be a selection bias during the course of the 12-month treatment duration. Subjects who experienced lack of efficacy did discontinue from the study; therefore, efficacy assessments at latter time points may be affected in part by this selection bias. No conclusions can be made regarding additional or further long-term efficacy.

## Conclusions

In this DO, open-label 1-year study, SDX/d-MPH was shown to be safe and well tolerated, with AEs that were comparable with those of other MPH products, with no new or unexpected safety findings. SDX/d-MPH also showed sustained efficacy during the 1-year treatment period.

## Clinical Significance

This is the second phase 3 trial of SDX/d-MPH in children aged 6–12 years that adds to the safety and tolerability data from the short-term pivotal laboratory classroom study. The results of this 1-year study showed that SDX/d-MPH was safe, well tolerated, and had continued efficacy during the 1-year treatment period.
